# High light stress triggers distinct proteomic responses in the marine diatom *Thalassiosira pseudonana*

**DOI:** 10.1186/s12864-016-3335-5

**Published:** 2016-12-05

**Authors:** Hong-Po Dong, Yue-Lei Dong, Lei Cui, Srinivasan Balamurugan, Jian Gao, Song-Hui Lu, Tao Jiang

**Affiliations:** 1 Research Center for Harmful Algae and Marine Biology, Key Laboratory of Eutrophication and Red Tide Prevention of Guangdong Higher Education Institutes, Jinan University, Guangzhou, 510632 China; 2 School of Ocean and Meteorology, Guangdong Ocean University, Zhanjiang, 524088 China

**Keywords:** *Thalassiosira pseudonana*, iTRAQ labeling, Quantitative proteomics, Light protection

## Abstract

**Background:**

Diatoms are able to acclimate to frequent and large light fluctuations in the surface ocean waters. However, the molecular mechanisms underlying these acclimation responses of diaotms remain elusive.

**Results:**

In this study, we investigated the mechanism of high light protection in marine diatom *Thalassiosira pseudonana* using comparative proteomics in combination with biochemical analyses. Cells treated under high light (800 μmol photons m^−2^s^−1^) for 10 h were subjected to proteomic analysis. We observed that 143 proteins were differentially expressed under high light treatment. Light-harvesting complex proteins, ROS scavenging systems, photorespiration, lipid metabolism and some specific proteins might be involved in light protection and acclimation of diatoms. Non-photochemical quenching (NPQ) and relative electron transport rate could respond rapidly to varying light intensities. High-light treatment also resulted in increased diadinoxanthin + diatoxanthin content, decreased Fv/Fm, increased triacylglycerol and altered fatty acid composition. Under HL stress, levels of C14:0 and C16:0 increased while C20:5ω3 decreased.

**Conclusions:**

We demonstrate that *T. pseudonana* has efficient photoprotective mechanisms to deal with HL stress. *De novo* synthesis of Ddx/Dtx and lipid accumulation contribute to utilization of the excess energy. Our data will provide new clues for in-depth study of photoprotective mechanisms in diatoms.

**Electronic supplementary material:**

The online version of this article (doi:10.1186/s12864-016-3335-5) contains supplementary material, which is available to authorized users.

## Background

Diatoms are unicellular, eukaryotic phytoplankton that account for approximately 40% of the marine primary productivity, which makes them indispensable for marine food webs [1]. Besides, they play a vital role in nutrient recycling and climate regulation [1]. In light of their ecological importance, the genomes of several diatom species have been sequenced [2], which greatly contributed to in-depth studies of diatom molecular biology. In the oceanic surface waters, diatoms are frequently exposed to drastic fluctuations in light intensity that can be harmful for photosynthesis and growth. For example, it is observed that in Big Island of Hawaii in July, maximum photosynthetically active radiation (PAR) ranged from 0 to 1300 μmol photons m^−2^s^−1^ on a clear day at a depth of 3 m while it ranged from 0 to 700 μmol photons m^−2^s^−1^ on a cloudy summer day [3]. Under such unpredictable and uncontrollable conditions, diatoms evolve sophisticated cellular mechanisms to protect the photosynthetic apparatus, which may be one of the reasons for their ecological success [4].

A number of physiological photoprotective mechanisms have evolved in various photosynthetic organisms to regulate photosynthesis under rapid light fluctuations. These mechanisms include the photosystem II (PSII) and PSI electron cycles, the fast repair of D1 protein of the PSII reaction center, the state-transitions, changes in the efficiency of energy shift from the harvesting complex to the reaction center, and non-photochemical quenching (NPQ) induced by activation of the xanthophyll cycle (XC) [5, 6]. Among these, the NPQ are of primary importance [7]. The XC constitutes the de-epoxidation of diadinoxanthin (Ddx) to diatoxanthin (Dtx) which is activated by the acidification of the thylakoid lumen resulting in accumulation of Dtx [7]. The levels of Ddx and Dtx were strongly affected by light intensity, and cells grown in high light (HL) almost doubled the pool size of XC pigments relative to cells grown in low light (LL) [8]. Silencing of the violaxanthin de-epoxidase gene in the diatom *Phaeodactylum tricornutum* resulted in reduced Dtx synthesis and NPQ, thus confirming the mechanistic model of the Dtx/NPQ relationship in diatoms [9]. In the ocean, XC and NPQ have shown to be the important features that might potentially influence niche adaptation of diatoms [10].

Molecular mechanisms underlying photoprotection in diatoms have been investigated in many studies. Eisenstadt and colleagues suggested that changes in the PSII core center play an important role in *P. tricornutum* during acclimation to varying light conditions [11]. Lhcx6 protein bound with Dtx could participate in heat dissipation of excess light energy under HL stress [12]. In *P. tricornutum,* D1 degradation rate as well as repair rate increased under HL exposure, suggesting the important role of D1 repair cycle in limiting photoinhibition [13]. The redox state of the PQ pool is considered to play a crucial role in the photosynthetic regulatory processes. A recent study revealed that high light acclimation in diatoms was triggered by the redox state of the plastoquinone pool [14]. More recently, chloroplast- localized death-specific protein (DSP1) was identified and its overexpression in *T. pseudonana* clone lines resulted in elevated cyclic electron flow (CEF) and expression of proteins involved in photosynthesis and carbon fixation [15]. However, these existing literatures have focused mainly on specific proteins rather than the whole algal proteome, therefore, the key genes and proteins involved in photoprotective responses to HL exposure in diatoms remain unknown.

“Omics” approaches are essential for reconstructing the metabolic pathways and regulatory networks responsible for light acclimation in diatoms. *T. pseudonana* is a centric diatom widely distributed throughout the world’s oceans. Its available genomic information, in combination with the key roles it plays in marine food webs and global carbon cycling, made it become model organism for molecular ecological studies. Several proteomic studies have been conducted in *T. pseudonana* to investigate the effects of nitrogen starvation and benzoapyrene exposure [16, 17]. A global regulation picture of the metabolic processes in response to nitrogen starvation was described, and some protein biomarkers were discovered when exposed to benzoapyrene. In addition, proteomic analysis in *T. pseudonana* under Fe limitation revealed that proteins involved in intracellular protein turnover pathways was increased, thus reducing demand for extracellular Fe [18]. In this study, we employ a proteomic approach based on isobaric tags for relative and absolute quantification (iTRAQ) labeling to examine the light protection mechanisms of *T. pseudonana* under excess light stress. This is the first proteomic study to demonstrate the protection strategies of a marine diatom to high light conditions experienced in the ocean. These data will provide new mechanistic insights into the responses of the marine diatom *T. pseudonana* to fluctuating light conditions from a proteomic perspective.

## Methods

### Growth conditions

Axenic *T. pseudonana*, obtained from the Provasoli-Guillard National Center for Marine Algae and Microbiota, was grown in artificial seawater supplemented with f/2 vitamins and inorganic nutrients [19]. Cultures were grown in growth chamber at 19 °C with irradiance of 30 μmol photons m^−2^s^−1^ (LL) and were kept in exponential growth phase for at least 4 weeks to ensure that all cells were acclimated. The illumination was provided by cool white fluorescent light with 12/12 h light/dark cycles. The growth rate was measured by monitoring in vivo fluorescence using a Turner Designs Model 10 Fluorometer (Turner Designs, CA, USA). For medium-light (ML, 200 μmol photons m^−2^s^−1^) and high-light (HL, 800 μmol photons m^−2^s^−1^) treatment, continuous light illumination was applied. The cells under LL conditions were divided into two parts. One part was exposed to 800 μmol photons m^−2^s^−1^ (HL) for 10 h for iTRAQ labeling, lipid and fatty acid analysis. The other part was kept under LL as a control. Samples for *Fv/Fm*, NPQ and pigment analysis were collected at time points 1, 3, 6, 10 and 12 h after transfer to ML or HL conditions. In a short-term experiment, cells were exposed to 1 h of ML or HL, followed by 1 h of LL recovery, with a subsequent 1 h ML or HL treatment, for determination of relative electron transport rate (rETR) and NPQ. Experiments were performed in triplicate for each treated and control cultures.

### Chlorophyll fluorescence measurements

The parameters *Fv/Fm* (the maximum photosynthetic efficiency of PSII) and rETR were determined using a Phyto-PAM Phytoplankton Analyzer (Walz, Germany). For *Fv/Fm*, 4 ml of the culture was dark-acclimated for 15–20 min before taking a measurement. NPQ was calculated using the Stern-Volmer parameter as NPQ = *Fm/Fm’* -1.

### Pigment analysis

For pigment analysis, 15 ml of culture was filtered using GF/F filter and the filter was immediately frozen in liquid nitrogen, and stored at −80 °C until analysis. Pigment quantitation was performed using an Agilent 1200 HPLC system (Agilent technologies, CA, USA) with a Symmetry C8 column (4.6 × 150 mm) following a previous method [20].

### Lipid and fatty acid analysis

For confocal microscopy analysis, cells were stained in the dark with Nile red at a final concentration of 1 μg ml^−1^ (from a stock of 0.1 mg ml^−1^ in acetone) and incubated in darkness, for 15 min. Images of oil bodies were captured using a LSM 510 META laser-scanning confocal microscope (Zeiss, Jena, Germany) with excitation wavelength of 488 nm and emission wavelength of 560–615 nm.

The neutral lipid content was detected using Nile red dye in combination with flow cytometry as described previously [21].

Fatty acids were analyzed directly from liquid cultures as described previously [22]. Lipids were extracted and derivatized from liquid culture. Fatty acid methyl esters (FAMEs) were analyzed by gas chromatography-mass spectrometry (GC-MS) in Analytical and Testing Center of Jinan University. FAMEs were quantified against a standard curve from different concentrations of C19:0 and C21:0 standard mixtures (nonadecanoic acid and heneicosanoic acid). The internal C19:0 and C21:0 standards run with each sample were used for calculating the recovery of FAMEs.

### Total protein extraction and iTRAQ labeling

Approximately 600–800 ml cultures were centrifuged at 8000 × g for 10 min at 4 °C. The supernatant was decanted, and cell pellets were lysed in rehydration buffer (7 M urea, 2 M thiourea, 10 mM dithiothreitol (DTT) and 4% W/V CHAPS) with sonication in ice using a microprobe (20% output, 20 to 30 cycles, 5 s each interrupted by 5 s of cooling). After centrifugation at 12,000 × g at 4 °C, the supernatants were reduced and alkylated with 10 mM DTT and 55 mM iodoacetamide (final concentration), respectively. The proteins in the supernatants were precipitated by adding 4 times volume of 100% cold acetone at −20 °C overnight, and the precipitates were resuspended in 0.8 M urea and 0.5 M tetraethylammonium bicarbonate (TEAB), pH 8.5. The protein concentration was measured using the Bradford method, thereafter digested with trypsin at 37 °C for 12 h. The tryptic peptides were labeled by the 8-plex iTRAQ reagents (AB Sciex, CA, USA) following the manufacturer’s protocol. After 2 h of labeling, the reaction solvents were evaporated by speed-vacuum, and the labeled peptides were dissolved in buffer A (25 mM NaH_2_PO_4_ in 25% acetonitrile, pH 2.7) for the peptide fractionation.

### Peptide fractionation by strong cation exchange (SCX) HPLC

SCX chromatography was performed with a LC-20AB HPLC Pump system (Shimadzu, Kyoto, Japan). The iTRAQ-labeled peptide mixtures were reconstituted with 4 ml buffer A and loaded onto a 4.6 × 250 mm Ultremex SCX column containing 5-μm particles (Phenomenex, USA). The peptides were eluted at a flow rate of 1.0 ml min^−1^ using a gradient of buffer A for 10 min, 5–60% buffer B (25 mM NaH2PO4, 1 M KCl in 25% acetonitrile, pH 2.7) for 27 min, and 60–100% buffer B for 1 min. The system was then maintained at 100% buffer B for 1 min before equilibrating with buffer A for 10 min prior to the next injection. Elution was monitored by measuring the absorbance at 214 nm, and fractions were collected every 1 min interval. The eluted peptides were pooled into 20 fractions, desalted with a Strata X C18 column (Phenomenex) and vacuum-dried.

### Peptide identification by nano RP HPLC and mass spectrometry

Each fraction was resuspended in buffer C (5% acetonitrile in 0.1% formic acid) and centrifuged at 20,000 × g for 10 min. Ten microliters of supernatant was loaded on a LC-20 AD nanoHPLC (Shimadzu, Kyoto, Japan) by the autosampler onto a 2-cm C18 trap column. Then, the peptides were loaded onto a 10-cm analytical C18 column (inner diameter 75 μm), with the flow rate at 8 μl min^−1^ in 4 min, and were eluted with the 35 min linear gradient starting from 2 to 35% B (95% acetonitrile in 0.1% formic acid) at 300 nl min^−1^, followed by a 5 min linear gradient to 60% B, which was followed by a 2 min linear gradient to 80% B.

Data acquisition was performed with a TripleTOF 5600 System (AB Sciex, Concord, CAN). The mass spectrometer was operated with an RP greater than or equal to 30,000 FWHM for TOF MS scans. For information-dependent acquisition (IDA), survey scans were acquired in 250 ms, and as many as 30 product ion scans were collected if exceeding a threshold of 120 counts per second (counts s^−1^). Four time bins were summed for each scan at a pulser frequency value of 11 kHz through monitoring of the 40 GHz multichannel TDC detector.

### Peptide and protein identification

The raw MS/MS data were converted into Mascot generic format (MGF), and the exported MGF files were searched by Mascot 2.3.02 (Matrix Science, London, UK) against the database with 24,591 predicted proteins in *T. pseudonana* downloaded from National Center for Biotechnology Information (NCBI) [23]. The search parameters were set as follows: tolerance of one missed cleavage of trypsin, oxidation (M) for methionine as the variable modifications, and carbamidomethyl (C) for cysteine, iTRAQ8-plex (N-term), and iTRAQ8-plex (K) as fixed modifications. A mass tolerance of 0.05 Da was permitted for intact peptide masses and 0.1 Da for fragmented ions. The charge states of peptides were set to +2 and +3. Specifically, an automatic decoy database search was performed to estimate the false discovery rate (FDR) of peptides in Mascot by choosing the decoy checkbox in which a random sequence of database is generated. The FDR was 1.1%. For peptide identification, only peptides at the 95% confidence interval (*P* < 0.05) by a Mascot probability analysis greater than “identity” were considered as confident.

### Quantitative data analysis for the iTRAQ labeling peptides

A unique protein with at least two unique peptides was qualified for further quantification data analysis. The quantitative protein ratios were weighted and normalized by the median ratio in Mascot. The peptide for quantification was automatically selected by Mascot to calculate the reporter peak area, error factor and *p*-value (default parameters in Mascot software package). The fold changes in protein abundance were defined as the median ratios of all significantly matched spectra with tag signals. Statistically significant variation (*P* < 0.05) and a ratio greater than 1.5 were used for cutoffs. The quantitation was performed at the peptide level following the procedures provided in http://www.matrixscience.com/help/quant_statistics_help.html. The student’s T-test was carried out using the Mascot software.

### Quantitative real-time PCR (qRT-PCR)

RNA was extracted using Trizol (Invitrogen). An equal amount of RNA was used for all samples to be transcribed into complementary DNA (cDNA) using the AMV First Strand cDNA Synthesis Kit. The resulting cDNA was diluted 8-fold as the qRT-PCR template. Amplicons were quantified in qRT-PCR reactions with ABI SYBR Green PCR Master Mix (Applied Biosystems). Additionally, qRT-PCR was performed in a LightCycler 480 instrument by standard methods (Roche) as previously described [24]. The 2^△△CT^ method was used to estimate fold changes in gene expression, normalized to the endogenous control gene, actin [24]: Comparative relative expression (2^△△CT^) = 2^– {[CT(HL) – CT(control)] – [CT(LL) – CT(control)]}^, where CT(HL) is the CT value of the target gene in a HL-treated sample, CT(LL) is the CT value of the same target gene in a LL-treated sample, and CT(control) is the CT value of the actin gene. Triplicate biological replicates were performed. The qRT-PCR primers are listed in Additional file 1: Table S1.

## Results and discussion

### Photosynthetic parameters and pigment analysis

**Fig. 1:**
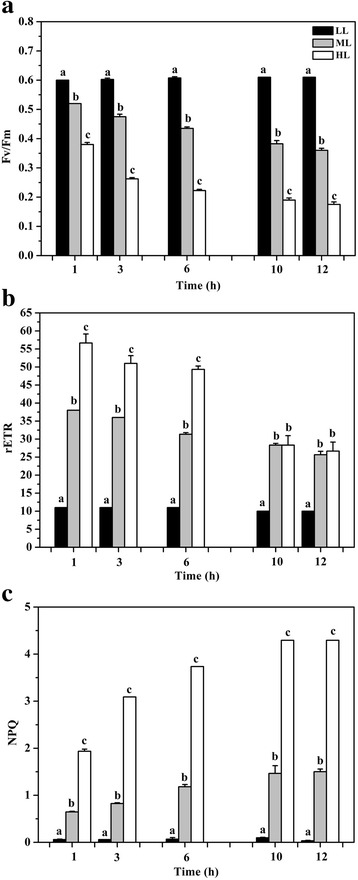
Time course of photosynthetic parameters after transition from low light (LL) to medium light (ML) or high light (HL). **a** Fv/Fm changes during 12 h of ML or HL treatment; **b** rETR changes during 12 h of ML or HL treatment; **c** NPQ changes during 12 h of ML or HL treatment. Different letters indicate significant differences between light regimes at *P* < 0.05. Error bars represent the standard deviations of the means generated from triplicates

**Fig. 2:**
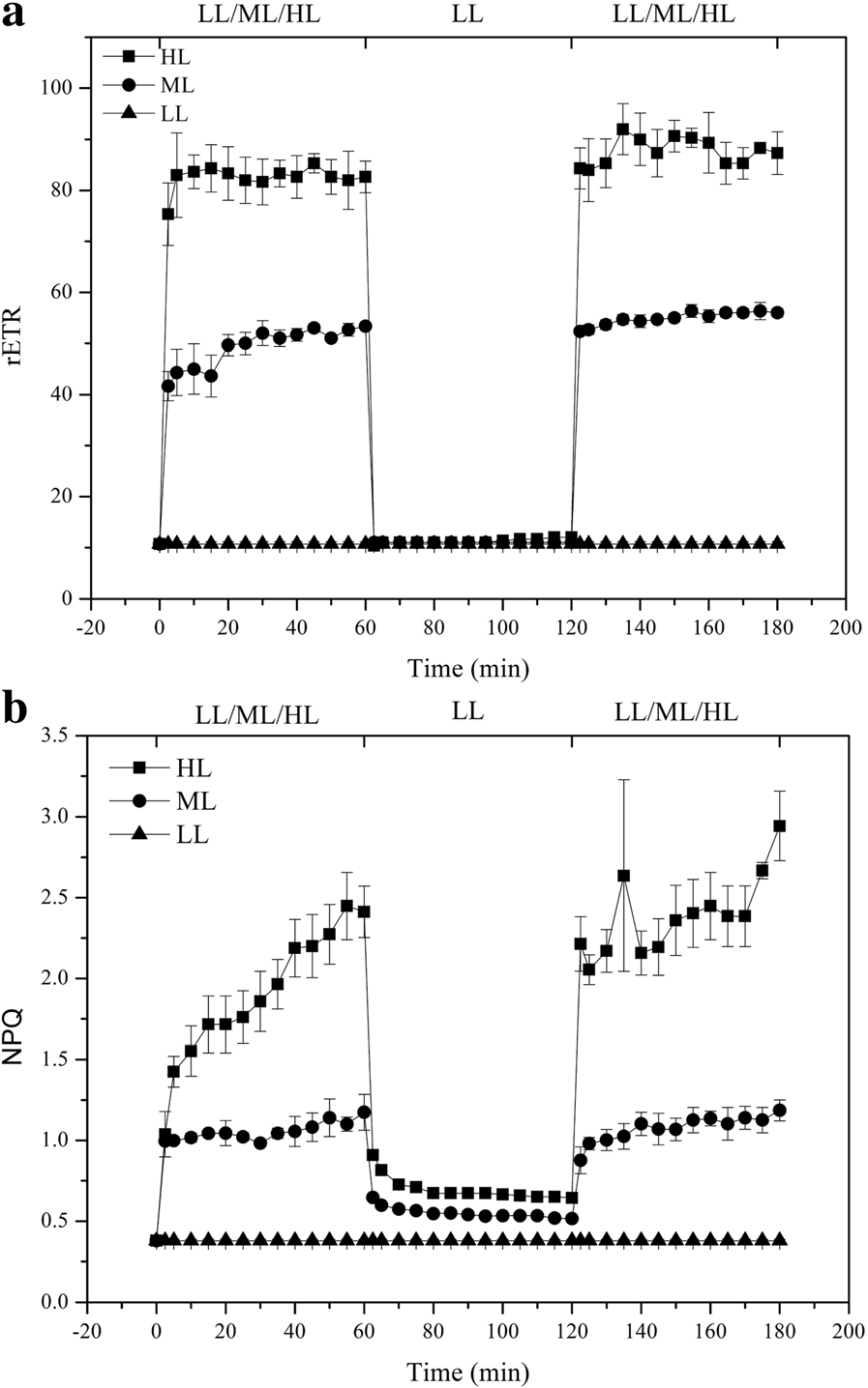
**a** rETR changes during 1 h of ML or HL treatment, 1 h of LL recovery, and subsequently 1 h of ML or HL treatment; **b** NPQ changes during 1 h of ML or HL treatment, 1 h of LL recovery, and subsequently 1 h of ML or HL treatment. Different letters indicate significant differences between light regimes at *P* < 0.05. Error bars represent the standard deviations of the means generated from triplicates

The maximum quantum yield of photosystem II (Fv/Fm) was reduced consecutively under HL or ML treatment exposed for 10 h (Fig. 1a) and almost did not change between 10 and 12 h ML or HL treatment. This trend has been observed in *P. tricornutum* [25] and in *T. pseudonana* [12]. rETR increased rapidly with increasing light intensities and maintained stable during 1 h of HL or ML treatment, thereafter decreased sharply when cultures were transferred to LL conditions (Fig. 2a). When LL-treated cells were exposed to longer ML or HL stress, a gradual and continuous decrease was observed in rETR over the 10 h time course (Fig. 1b). These data suggest that photosystem II and electron transport chain in cells are damaged to some extent during long-term HL stress.

NPQ is an important photoprotective mechanism that safely dissipates excess energy as heat. In our study, NPQ increased consecutively during 1 h of ML or HL treatment, decreased sharply during 1 h of LL recovery and increased once again during subsequently 1 h of ML or HL treatment (Fig. 2b). With longer exposure to ML or HL, the level of NPQ was increased continuously during 10 h, but almost remained stable after 10 h ML or HL (Fig. 1c). In addition, NPQ was much higher under HL treatment than under ML treatment. These findings indicate that both the ML and HL can induce photoprotective responses in *T. pseudonana*. The increase of NPQ in *T. pseudonana* under HL treatment has been reported previously [12]. But, there were the difference in the highest NPQ value in these two studies. This may be due to the growth characteristics of cells, culture conditions, light intensity and instrument difference.

**Fig. 3:**
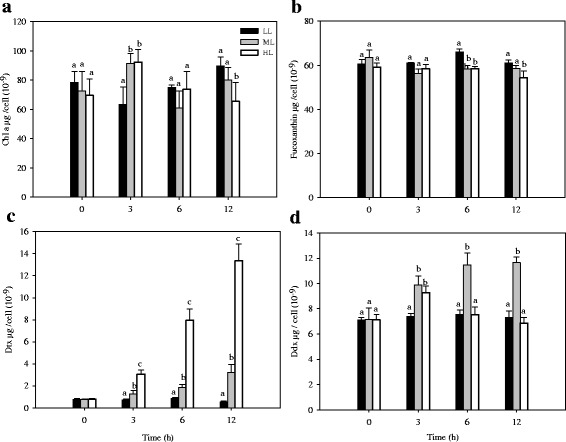
The Chl *a* (**a**), fucoxanthin (**b**), Dtx (**c**) and Ddx (**d**) contents per cell as a function of medium light (ML) or high light (HL) exposure time. Cultures were grown exponentially at a light intensity of 30 μmol photons m^−2^s^−1^ for 4 weeks prior to HL treatment. Error bars represent the standard deviations of the means from three biological replicates. Different letters indicate significant differences between light regimes at *P* < 0.05

HPLC analyses showed that chlorophyll *a* (Chl *a*), fucoxanthin, Ddx and Dtx were the dominant pigments in this alga (Fig. 3). Compared to LL, Chl *a* and fucoxanthin content did not change considerably during 12 h of ML or HL stress (Fig. 3a and b). In contrast, there was a gradual and continuous increase in Dtx concentration (Fig. 3c). Meanwhile, the Dtx content per cell increased with increasing light intensities, and the Dtx content in cultures under HL treatment was 2 to 3 times that of Dtx in cultures under ML treatment over the culture period (Fig. 3c). The results indicate that Dtx accumulation plays a vital role in photoprotection of diatoms. It has been shown that increased Dtx is vital for maintaining high level of NPQ during prolonged HL treatment [12].

As far as Ddx was concerned, its content showed a drastic change under ML or HL stress. Under ML stress, Ddx content in the culture increased markedly in the first 6 h, whereas it fluctuated under HL stress over the whole course of the experiment (Fig. 3d). Compared to LL, the Ddx content was upregulated significantly in cultures exposed to ML for 3, 6, and 12 h, but it was just elevated in cultures exposed to HL for 3 h (Fig. 3d). These data indicate that excess light treatment elicits *de novo* synthesis of the Dtx + Ddx pool. It is likely that increased Dtx + Ddx pool consume partial excess light energy. It has also been shown that the *de novo* synthesis of Dtx + Ddx could enhance the antioxidant activity within the thylakoid membranes during prolonged high-light stress [14]. In a previous study on *T. pseudonana*, Dtx content per cell gradually increased while there was slight change in Ddx content during 9 h of HL treatment [12]. Our results support the conclusion that the accumulation of Dtx is not from the conversion of Ddx during long-term HL stress [12].

### Changes of fatty acid composition and neutral lipid content

**Fig. 4:**
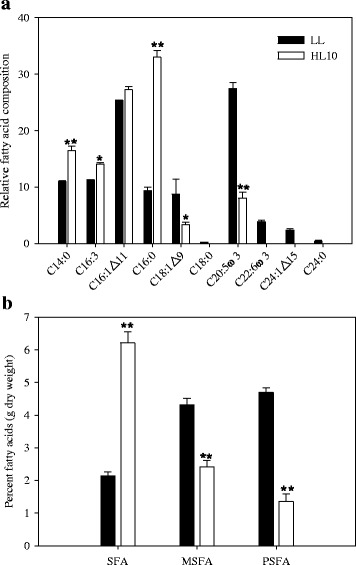
Changes in fatty acid composition in *T. pseudonana* after 10 h of high light (HL) treatment. **a** Relative fatty acid composition (percentage of total fatty acids); **b** total fatty acids (TFA), saturated fatty acids (SFA), monounsaturated fatty acids (MSFA) and polyunsaturated fatty acids (PSFA). Low light (LL)-acclimated cells were shifted into HL and sampled at 10 h. * indicates significant differences (*P* <0.05) relative to LL-acclimated cells

It has been shown that excess light energy might increase neutral lipid content in marine diatom *Skeletonema marinoi* [26], which further affect the growth of zooplankton in the marine food chain. Here, we analyzed fatty acid composition and neutral lipid content in *T. pseudonana* under HL stress. After 10 h of HL stress, saturated fatty acid content increased significantly (Fig. 4b), whereas monounsaturated and polyunsaturated fatty acid contents decreased significantly. As shown in Fig. 4a, the dominant fatty acids present in the fatty acid profile were C14:0, C16:3ω3, C16:1△11, C16:0, C18:1△9, C20:5ω3, and C22:6ω3 (number of carbons: number of double bonds, where positions of double bonds are indicated with △ [counting from the carboxyl group] or ω [counting from the methyl group]). Among the alterations in fatty acid composition (%) within the total fatty acids, it was observed that levels of C14:0 and C16:0 were markedly increased under HL, while no significant change in C18:0 and C24:0 was observed. In addition, C18:1△9, C20:5ω3 and C22:6ω3 were significantly decreased after HL stress. The level of C20:5ω3 was reduced by approximately 70%, and C22:6ω3 was below the detection limit. The level of C16:3ω3 was found to increase after HL stress. Interestingly, C16:1△11 increased slightly after HL stress. These data indicated that HL stress altered the fatty acid composition. This alteration may raise the capability to resist excess light stress. Similar to N depletion [27], increased saturated fatty acid synthesis may alleviate reactive oxygen species formation at PSII by sequestering excess electrons moving through the photosynthetic electron transport chain.

**Fig. 5:**
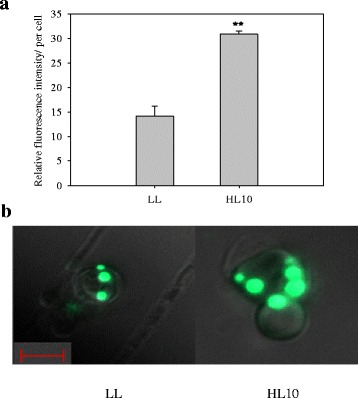
Accumulation of TAGs during 10 h of high light (HL) stress. **a** TAG content determined by Nile red staining; **b** confocal microscopy images of Nile red-stained cells subjected to HL treatment. * indicates significant differences (*P* <0.05) relative to low light (LL)-acclimated cells. The scale bar indicates 5 μm

Surprisingly, the neutral lipid content (triacylglycerol, TAG) determined by Nile red fluorescence staining showed that neutral lipid increased significantly after HL treatment (Fig. 5a). In addition, a significant increase was observed in size and number of lipid droplets in a typical diatom cell (Fig. 5b). These data suggest that HL stress elicits TAG accumulation in diatom cells. In microalgae, TAG accumulation under environmental stress conditions may be associated with plastid membrane turnover or degradation [28]. Thus, it is hypothesized that the membrane glycerolipids with polyunsaturated fatty acids may be degraded to produce additional TAG during HL treatment.

### Proteomic responses to HL treatment

Based on the data above, it has been shown that HL stress lead to significant changes in diatom physiology. Considering that diatoms are frequently subjected to varying light irradiance in surface waters of ocean, HL were used as irradiance condition to examine the effect of excess light on the proteome of *T. pseudonana*. Transcriptional studies indicate that the acclimation mechanisms in diatoms can be divided into an initial response phase (0–0.5 h), an intermediate acclimation phase (3–12 h) and a late acclimation phase (12–48 h) [25]. A strong and rapid regulation of genes encoding proteins involved in photosynthesis, pigment metabolism, reactive oxygen species (ROS) scavenging systems, Dtx/Ddx cycle, and carbon metabolism is observed during 12 h of HL treatment. It is inferred that proteomic responses should be later than transcriptional changes, thus 10 h of HL treatment was chosen to track short-term photoprotective mechanisms of diatoms. The duration may reflect the changes after diatom cells are subjected to a daytime irradiance in the oceanic surface waters.

#### Protein identification, quantification and qRT-PCR verification

Protein samples from LL-acclimated and HL-treated cultures were labeled by six different iTRAQ tags (113, 115, 121, 114, 116, and 118). Each treatment consisted of three biological replicates. Detailed interpretation procedure of protein labeling and identification are described in Additional file 2: Figure S1. After data search by Mascot, a total of 4143 proteins were identified with one or more unique peptides (Additional file 3: Table S2). Among them, approximately 66% of total proteins were matched by two or more unique peptides. The identified proteins were annotated by blasting against UniProt/Swiss-Prot, Gene Ontology (GO) and Cluster of Orthologous Groups (COG) databases. As a result, about 65% of proteins could be assigned to a detailed function. Protein quantification was performed for these proteins matched by two or more unique peptides.

For protein quantification, repeatability among three biological replicates was analyzed (Additional file 4: Figure S2). Error distribution showed that median error was from 0.085 to 0.12 for the three biological replicates, and approximately 75 to 78% of proteins varied within 20% of median value, which is a reasonable range for shotgun proteomics. Based on the error distribution among replicates, we chose >1.5- or <0.67-fold change as the threshold of differentially expressed proteins. Following the criteria, 132 and 11 were upregulated and downregulated, respectively, under HL stress (Additional file 5: Table S3). These proteins were classified based on GO function (Table 1). Proteins that were involved in protein synthesis/degradation, photosystem and electron transport chain, and photoreceptor and signal were predominant in GO categories.

**Table 1:** Number and percentage of the differentially expressed proteins (DEP) grouped by Gene Ontology

GO group	Number of DEP in HL compared to LL	% of total DEP
Carbon fixation	2	1.4
Xanthophyll cycle	1	0.7
Photosystem and electron transport chain	12	8.4
Photoreceptor and signal	12	8.4
Carbon metabolism	4	2.8
Protein synthesis/degradation	35	24.5
Fatty acid, steroid and terpenoid synthesis	7	4.9
Oxidative stress	8	5.6
Cell cycle and cell division	2	1.4
DNA/RNA replication, modification and repair	4	2.8
Energy production	2	1.4
Nitrogen metabolism	2	1.4
Transport	6	4.2
CO2 concentration	1	0.7
Cell wall and extracellular enzyme	4	2.8
No GO	14	9.8
Function unknown	27	18.9

**Fig. 6:**
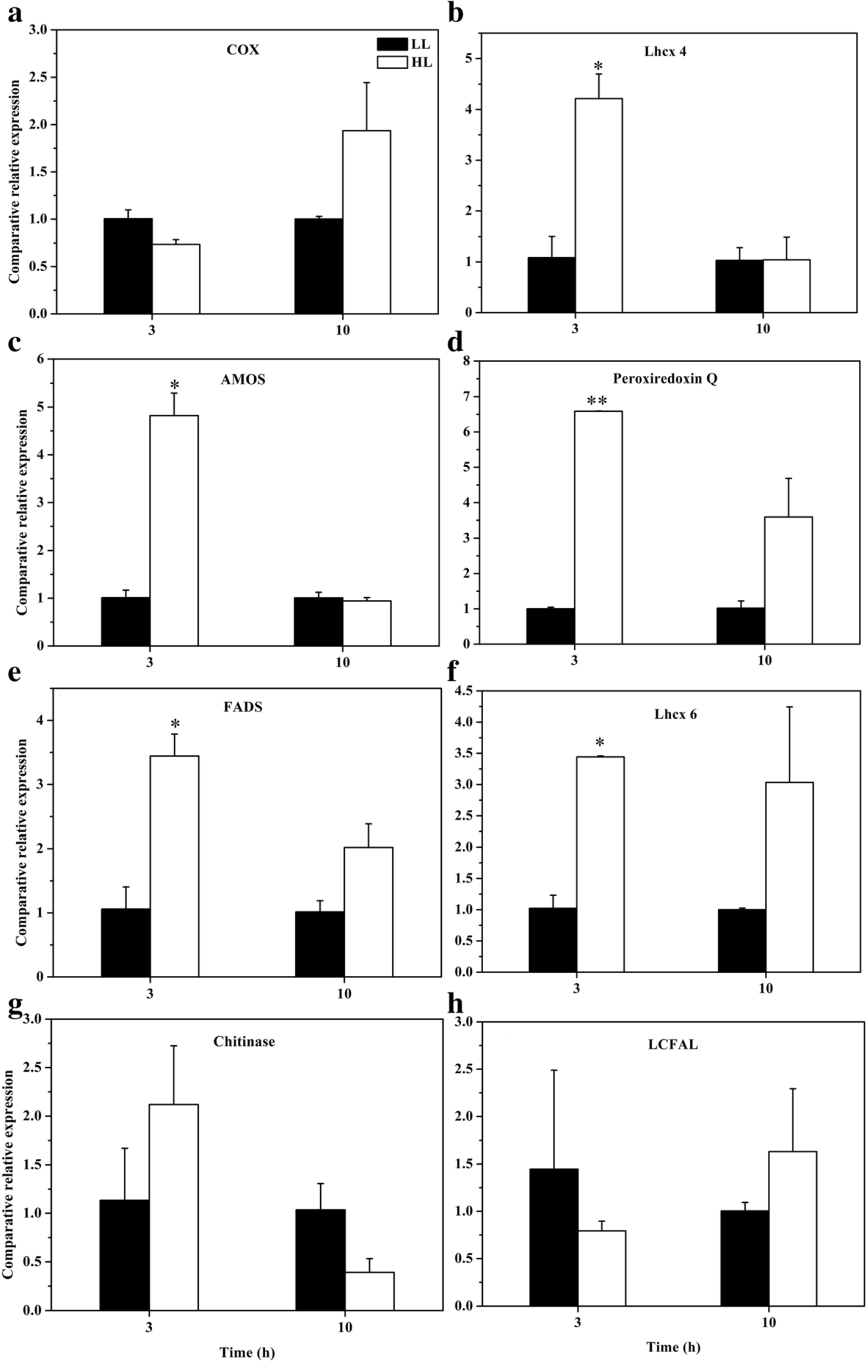
Comparative relative expression (△△CT) patterns of genes **a** COX, **b** Lhcx 4, **c** AMOS, **d** peroxiredoxin Q, **e** FADS, **f** Lhcx 6, **g** chitinase, **h** LCFAL encoding eight differentially expressed proteins identified in the proteomic study when low light (LL)-acclimated cells were exposed to high light (HL) for 3 and 10 h. Error bars represent the standard deviations of the means generated from triplicates. *indicates significant differences (*P* <0.05) relative to LL-acclimated cultures. The abbreviations are as follows: glycolate oxidase, GOX; antibiotic biosynthesis monooxygenase, AMOS; fatty acid desaturase, FADS; long-chain-fatty-acid-CoA ligase, LCFAL

The transcript abundance of genes encoding eight upregulated proteins was determined by qRT-PCR (Fig. 6). Among these genes, expression levels of five genes including Lhcx4, antibiotic biosynthesis monooxygenase (AMOS), peroxiredoxin Q, fatty acid desaturase (FADS), and Lhcx6 were markedly upregulated after cells were exposed to HL for 3 h, but were almost back to LL levels after 10 h HL (Fig. 6b, c, d, e and f). The data demonstrated that the five genes could be induced by short-term HL stress and supported indirectly proteomic results. For other three genes, glycolate oxidase, chitinase, long-chain-fatty-acid-CoA ligase (LCFAL), no significant difference was observed in their transcript abundance after cells were exposed to HL for 3 and 10 h (Fig. 6a, g and h). It is assumed that they might be regulated at post-transcriptional or translational or post-translational level.

#### Light-harvesting complex (LHC) and XC

Diatoms possess a large number of members of LHC superfamily, the fucoxanthin Chl *a/c* proteins (FCPs). These FCPs fall into three groups: the major fucoxanthin Chl *a/c* proteins (Lhcf), the red algal-like proteins (Lhcr) and the LI818-like proteins (Lhcx). Interestingly, in PSI and PSII, only FCPs were differentially regulated by HL. After 10 h of HL treatment, expression of 4 FCPs, Lhcx4, Lhcx6, Lhcr5 and Lhcr8, were increased by 2.1- to 6.3-fold and no FCPs decreased (Table 2). The results suggest that Lhcx4, Lhcx6, Lhcr5 and Lhcr8 are involved in photoprotection in *T. pseudonana* during HL stress. These FCPs may bind to Dtx and play a role in photoprotection. The Lhcx6 protein from *T. pseudonana* has been shown to participate in heat dissipation of excess light energy during HL stress [12]. Interestingly, a violaxanthin de-epoxidase-like (VDL) increased by 1.64-fold after 10 h of HL treatment (Table 2). It is deduced that the VDL is involved in the conversion of Ddx to Dtx under HL.

**Table 2:** The differentially expressed proteins identified in *T. pseudonana* under 10 h of HL which are involved in important metabolic pathways disscussed in this study

Name	Accession number	Number of unique peptides	Fold change (HL/LL)
Light-harvesting complex proteins and xanthophyll cycle			
Fucoxanthin chl a/c light-harvesting protein (Lhcr8)	gi|220969130	4	2.04
Fucoxanthin chlorophyll a/c light-harvesting protein (Lhcr5)	gi|209583680	6	3.64
Fucoxanthin chlorophyll a/c protein, LI818 clade (Lhcx6)	gi|220969145	5	3.84
Fucoxanthin chl a/c light-harvesting protein (Lhcr8)	gi|220968241	4	2.32
Fucoxanthin chlorophyll a/c protein, LI818 clade (Lhcx4)	gi|220974177	2	6.34
Violaxanthin de-epoxidase-like 1	gi|220974348	3	1.64
Carbon metabolism			
Ribulose-1,5-bisphosphate carboxylase/oxygenase large subunit	gi|4093213	2	1.83
Phosphoribulokinase	gi|220970595	4	1.95
Carbonic anhydrase	gi|589908190	3	2.88
Phosphoglycerate kinase	gi|220968544	10	1.67
Glycolate oxidase	gi|220975250	4	2.26
Chitinase	gi|220967856	8	1.62
Peptidoglycan-associated outer membrane protein	gi|220969879	2	1.75
Antibiotic biosynthesis monooxygenase	gi|220968047	2	5.14
Lipid metabolism			
Long-chain-fatty-acid-CoA ligase	gi|209585777	3	1.61
Fatty acid desaturase	gi|220973009	6	2.21
Cyclopropane-fatty-acyl-phospholipid synthase	gi|220969789	4	1.98
Serine palmitoyltransferase	gi|220968852|	4	1.68
Omega-6 fatty acid desaturase	gi|220975284	3	2.14
Nucleoside-diphosphate-sugar epimerase	gi|220967789	11	1.59
Signaling proteins and transcription factors			
Retinol dehydrogenase	gi|220978263	7	1.68
Retinol dehydrogenase	gi|220973743	4	4.39
Death-specific protein 1	gi|156600453	2	2.26
Nucleoprotein TPR	gi|220969246	16	1.63
EH domain-containing protein	gi|220977976	7	2.69
ATP-dependent RNA helicase ded-1	gi|220972152	7	2.46
Uncharacterized aarF domain-containing protein kinase At4g31390	gi|220969732	7	1.61
Ran-type small G protein	gi|220976883	4	1.50
EF-Hand 1, calcium-binding site	gi|220976291	3	2.01
U6 snRNA-associated Sm-like protein LSm7	gi|220969887	2	2.07
Oxidative stress			
Peroxiredoxin Q, chloroplastic	gi|220975768	2	7.83
Peptide methionine sulfoxide reductase MsrA	gi|220976092	5	2.2
Thiol-disulfide isomerase	gi|220969193	11	1.84
Co-chaperonin GroES (HSP10)	gi|220971585	4	2.13
Heat shock protein DnaJ	gi|220977601	12	1.76
Chaperone protein dnaJ	gi|220971047	7	1.77
high light induced protein 2	gi|220977122	2	1.57
Stress-inducible protein sti1	gi|220969212	6	1.78

#### Carbon metabolism

We identified two ribulose-1,5 bisphosphate carboxylase/oxygenases (Rubisco) involved in the Calvin-Benson cycle in the proteome of *T. pseudonana.* Among them, a Rubisco increased by 1.83-fold in cells during 10 h of HL treatment (Table 2), whereas another Rubisco did not change. In addition, a phosphoribulokinase (PKK) that is involved in the Calvin-Benson cycle increased by 1.95-fold in HL-treated cells (Table 2). It is inferred that the Calvin cycle may be elevated in HL-treated cells relative to LL-acclimated cells.

Out of two gamma carbonic anhydrases (CA) identified, one increased by 2.88-fold after 10 h of HL treatment (Table 2). In addition, two of phosphoenolpyruvate carboxylases (PEPCase), one phosphoenolpyruvate carboxykinase (PEPCK), one pyruvate phosphate dikinase (PPDK) which are required for CO_2_-concentrating mechanisms (CCM) of C4-metabolism were identified in our study and they were not affected after HL treatment. The genes encoding PEPCase, PEPCK and PPDK have been identified in *T. pseudonana* genome [29]. Our data confirm the presence of CCM in *T. pseudonana*. It has been shown that high light irradiance increased CA activity, thus recycled CO_2_ leaking out of the chloroplast more effectively [30]. Thus, the CA upregulation suggested elevated CCM in HL-treated cells. The elevated CCM in C4-metabolism might increase consumption for cellular energy [30, 31].

It is noted that a phosphoglycerate kinase (PGK) increased by 1.67-fold in cells experiencing 10 h of HL treatment (Table 2). The PGK were predicted to be localized in the cytoplasm and this clue suggests that the glycolysis pathway in cytoplasm may be accelerated after HL treatment. A glycolate oxidase (GOX) that is a key enzyme of the photorespiration/glyoxylate cycle, increased by 2.3-fold after 10 h of HL treatment (Table 2). In higher plants, photorespiration can reduce the damage caused by the oxygenation reaction of Rubisco. When O_2_ reacts to ribulose-1,5-bisphosphate, one molecule of 2-P-glycolate and one molecule of 3-P-glycerate are produced. The former is degraded via the photorespiratory pathway, which avoids its inhibition for the Calvin cycle enzyme, triosephosphate isomerase. It is likely that photorespiration activity increased after short-term HL treatment. It suggested that diatoms may alleviate HL stress by releasing more fixed carbon [32].

Several enzymes that are involved in synthesis and degradation of chitin were identified in *T. pseudonana,* including chitin synthetase, chitin deacetylase, and chitinase. Cell wall chitin content in diatoms was found to affect sinking of diatoms in the surface layers of the ocean [33]. Diatom sinking is proposed to be a survival strategy for cells in harsh environments and enables them to stay in favorable conditions [34]. In this study, our data confirmed the presence of chitin in diatoms and found that a chitinase was increased by 1.6-fold following 10 of HL treatment, whereas there was no change in chitin synthetase level (Table 2). This result implies that short-term HL may elicit a decrease in cell wall chitin in diatom. We also identified a peptidoglycan-associated outer membrane protein (PPOM) and an AMOS which were increased by 1.75- and 5.14-fold, respectively, after 10 h of HL treatment (Table 2). They were predicted to be localized in cell wall and involved in synthesis of polysaccharides. The data suggest that the synthesis of polysaccharides in diatom cell walls may be elevated after HL treatment. The hydrophilic nature of polysaccharides probably fortifies the stability and rigidity of cell walls [35], which helps the diatoms to resist HL stress.

#### Lipid metabolism

We identified five enzymes involved in the synthesis of fatty acids or lipids that increased by 1.61- to 2.21-fold after 10 h of HL treatment, including LCFAL, FADS, cyclopropane-fatty-acyl-phospholipid synthase (CFAPS), serine palmitoyltransferase (SRPS) (Table 2). In higher plants, the synthesized fatty acid in the plastid is converted to acyl-CoA by long-chain fatty acid CoA ligase (LCFAL) [36], which is subsequently used for synthesis of TAG and phospholipids. CFAPS is involved in synthesis of phospholipid. In addition, a nucleoside-diphosphate-sugar epimerase (NDSE) that is involved in UDP-galactose supply for galactolipid biosynthesis of thylakoid membranes in rice chloroplasts [37] was found to be upregulated by 1.59-fold after 10 h of HL treatment (Table 2). Overexpression of NDSE gene in rice was reported to increase photosynthetic efficiency, biomass, and grain production [37]. It is assumed that upregulation of LCFAL, CFAPS, and NDSE may increase TAG in cells, phospholipids, and galactolipids in thylakoid membranes, which may elevate utilization efficiency of light energy and strengthen membrane stability under HL stress. Increased TAG has been observed in the physiological studies mentioned above. The SRPS is a key enzyme in the biosynthesis of sphingolipids [38] and its upregulation suggests that cellular sphingolipids content may be increased after HL stress. Plant sphingolipids have been shown to be involved in signal transduction, membrane stability, and stress responses [38]. Interestingly, two of FADSs increased by 2.14- to 2.21-fold after HL treatment. FADSs catalyze key reactions in the synthesis of polyunsaturated fatty acids. It is likely that FADSs may be responsible for the increased percentage of C16:3ω3 and C16:1△11 within total fatty acids.

#### Photoreceptors, signaling proteins, and transcription factors

We identified three aureochromes, which have been regarded as photoreceptors in *P. tricornutum* and *T. pseudonana* [39]. Under HL stress, no significant change was observed in relative abundance of aureochromes, suggesting that light signal transmission might be not regulated by change of expression abundance of aureochromes. In addition, five putative retinol dehydrogenases (RDS) were identified in *T. pseudonana* and two were shown to be upregulated by 1.68- and 4.39-fold, respectively, after 10 h of HL treatment (Table 2). RDS is responsible for the conversion of retinol to retinal in the visual cycle, which is the chromophore of the photoreceptor rhodopsin. Identification and changes in RDS suggest the likely presence of homologous proteins with rhodopsin in diatoms. In addition, upregulation of RDSs under HL treatment suggests that they may be involved in transmission of light signal in cells.

Eight signaling proteins or transcription factors were found to be differentially expressed after HL treatment (Table 2), including ran-type small G protein, DSP1, U6 snRNA-associated Sm-like protein LSm7, nucleoprotein TPR, EH domain-containing protein, and ATP-dependent RNA helicase ded-1. These proteins may allow light signals from photoreceptors to trigger nuclear gene expression that facilitate various cellular processes. Interestingly, the DSP1 has been identified in *T. pseudonana* in a previous study, which showed that DSP1 localizes to the plastid and that clone lines overexpressing DSP1 had increased CEF around photosystem I [15]. In this study, upregulation of DSP1 may increase the flux of electrons through CEF, thus reducing electrons produced by excess light.

#### Nitrogen metabolism

Interestingly, 21 to 25 ribosomal proteins that make up the ribosomal subunits were upregulated significantly after 10 h of HL (Table 3 and Additional file 5: Table S3). Three translation initiation factors were shown to be upregulated by 1.6- to 1.9-fold after 10 h of HL treatment (Table 3). Among them, five ribosomal proteins and an IF-2 were localized to the chloroplasts. Therefore, it is deduced that protein synthesis might be elevated in cytoplasm and chloroplast after HL treatment. This is in agreement with the observation that 132 proteins showed upregulation and 11 downregulation after HL treatment. A Tha4/Hcf106 protein was shown to be upregulated by 3.0-fold after HL treatment (Table 3). This protein is involved in the thylakoid ΔpH-dependent pathway that transports nuclear-encoded thylakoid proteins into chloroplast stroma [40]. Upregulation of Tha4/Hcf106 suggests that more thylakoid-bound precursor proteins were transported into the chloroplasts after HL stress.

**Table 3:** The differentially expressed proteins identified as involved in nitrogen assimilation, transport and protein synthesis in *T. pseudonana* under 10 h of HL stress

Name	Accession number	Number of unique peptides	Fold change
Glutamine synthetase	gi|220969384	11	1.53
Ornithine cyclodeaminase	gi|220977545	5	1.99
Urea transporter	gi|220971788	4	1.64
ABC transporter	gi|220977750	5	2.56
ABC transporter	gi|220975701	26	1.65
ABC transporter	gi|220968000	4	1.78
Tha4/Hcf106 protein	gi|220977108	3	2.98
30S ribosomal protein S11, chloroplastic	gi|125987672	2	4.69
60S ribosomal protein L27a	gi|220976691	4	1.69
Translation initiation factor eIF-2	gi|209586387	16	1.64
40S ribosomal protein S18	gi|220976243	5	3.3
40S ribosomal protein S20	gi|220974843	3	2
Translation initiation factor 1	gi|209585886	3	1.62
50S ribosomal protein L5, chloroplastic	gi|224493190	7	2.11
60S ribosomal protein L13	gi|220974751	4	1.56
40S ribosomal protein S25	gi|220970481	2	2.46
40S ribosomal protein S11	gi|220973471	8	2.04
60S ribosomal protein L27	gi|220970128	7	2.26
30S ribosomal protein S6, chloroplastic	gi|220967765	8	1.93
60S ribosomal protein L12-1	gi|220976821	4	2.57
60S ribosomal protein L35a	gi|220971603	4	2.16
60S ribosomal protein L24	gi|220975456	4	1.48
40S ribosomal protein S26-B	gi|220973256	2	2.45
60S ribosomal protein L6	gi|220970841	9	1.99
40S ribosomal protein S9	gi|220974914	5	1.62
60S ribosomal protein L23a	gi|220973318	6	2.65
60S ribosomal protein L23	gi|220971079	6	1.72
60S ribosomal protein L19	gi|220976636	2	2.39
60S ribosomal protein L11	gi|220974977	5	2.03
Translation initiation factor IF-2	gi|220974959	4	1.88

#### General stress

Three enzymatic antioxidants were upregulated by 1.8- to 7.8-fold after 10 h of HL treatment, including chloroplastic peroxiredoxin Q, thiol-disulfide isomerase and peptide methionine sulfoxide reductase (MsrA) (Table 2, Additional file 5: Table S3). In *P. tricornutum,* a peroxiredoxin Q gene was among the strongest and the most consistently upregulated genes during HL treatment [25]. It has been suggested that peroxiredoxin Q from *A. thaliana* was involved in the photosystem II protection against H_2_O_2_ [41]. Our study demonstrates that peroxiredoxin Q is vital for the normal growth of *T. pseudonana* in HL conditions on the ocean surface or during pool cultivation. The thiol-disulfide isomerase was first found in *Emiliania huxleyi* and is a novel selenoprotein, which is homologous to protein disulfide isomerase (PDI) and contains a highly conserved thioredoxin domain [42]. It is the second selenoprotein found in *T. pseudonana* in addition to sec-containing glutathione peroxidase [43]. MsrA catalyzes the reduction of methionine sulfoxide to methionine, which protects methionine in proteins from being oxidized [44]. Upregulation of MsrA suggests that MsrA plays an important role in protecting cellular proteins against photooxidation. Interestingly, we identified a high light induced protein 2 (ELIP) which was upregulated by 1.57-fold after 10 h of HL stress. In higher plant, ELIPs belong to the family of light-harvesting complexes (LHCs), but they differ from the LHCs in response to HL stress. The LHCs bind chlorophyll and absorb solar energy and their expression is inhibited by HL. In contrast, ELIPs have been shown to be involved in photoprotection by binding chlorophyll released during turnover of LHCs or stabilizing LHCs during HL stress [45]. Our data confirmed the presence of ELIPs in marine diatoms.

Polypeptides will become misfolded or unfolded when cells are subjected to stress of adverse conditions. Heat shock proteins (HSP) can prevent misfolded proteins from aggregation in the cells and help them refold. Six HSP-related proteins were found to be upregulated significantly after 10 h of HL treatment, including chaperone protein dnaJ (CDnaJ), stress-inducible protein (STI1), heat shock protein DnaJ (HDnaJ), and co-chaperonin GroES (HSP10) (Table 2). STI1 functions as a co-chaperone which reversibly links HSP70 and HSP90 together and regulates the activities of the linked proteins [46]. In cyanobacteria and *Arabidopsis*, HL and H_2_O_2_, respectively, induced chaperones and HSPs [47], suggesting that certain HSPs are involved in protection function against oxidative conditions. Therefore, in *T. pseudonana*, the increased expression of HSP-related proteins may be an adaptable response to reduce the protein aggregation and misfolding caused by reactive oxygen species (ROS) during excess light treatment.

## Conclusions

Using a quantitative proteomic approach based on iTRAQ labeling of peptides in combination with biochemical analyses, we studied the responses of diatoms to HL stress. We have demonstrated that light-harvesting complex, ROS scavenging systems, photorespiration/glyoxylate cycle, lipid metabolism and protein synthesis were involved in responses of diatoms to HL stress. Proteomic changes elicit a series of physiological changes in diatoms. Under HL stress, Fv/Fm decreased and NPQ increased; the size of Dtx + Ddx pool increased; TAG increased and fatty acid composition was altered. Interestingly, certain specific proteins might fulfill a photoprotective function, including Lhcx6, Lhcx4, Lhcr8, Lhcr5, enzymatic antioxidants, ELIPs, and HSP-related proteins. These proteins protect diatom cells from photooxidation. In addition, marked upregulation of RDS probably suggests that other than the Dtx/Ddx cyle, the conversion of retinol to retinal may contribute to dissipation of excess light energy. These data will provide new insights into protection and acclimation strategies of diatoms in response to varying light conditions.

## Additional files

Additional file 1: Table S1. Primers used in this study for qRCR. (DOC 40 kb)
Additional file 2: Figure S1. Workflow for proteomic experiments using iTRAQ labeling in this study. *T. pseudonana* cells were acclimated in low light (LL) for 4 weeks and exposed to high light (HL) for 10 h. Three biological replicates were performed. The extracted proteins were reduced, alkylated, and digested with trypsin after cell pellets were lysed. The tryptic peptides were then labeled with iTRAQ reagents and put together as shown. The SCX chromatography was performed to fractionate peptides and fractions were collected. The eluted peptides were analyzed by LC-MS/MS. (PPT 61 kb)
Additional file 3: Table S2. List of proteins identified with by MS/MS from the T. pseudonana extract with a minimum of 1 peptides at >95% confidence in high light (HL)-treated experiment. (XLS 1082 kb)
Additional file 4: Figure S2. Error distribution (A, B, C, D, E, and F) among three biological replicates and CV distribution (G) between control and HL-treated samples. (DOC 250 kb)
Additional file 5: Table S3. The significantly differentially expressed proteins regulated by high light (HL) (Fold change > 1.5 or < 0.67, *P* < 0.05). (XLS 72 kb)

## Abbreviations

AMOS: Antibiotic biosynthesis monooxygenase
CA: Carbonic anhydrases
CCM: CO_2_-concentrating mechanisms
CDnaJ: Chaperone protein dnaJ
CEF: Cyclic electron flow
CFAPS: Cyclopropane-fatty-acyl-phospholipid synthase
Chl *a*: Chlorophyll *a*
COG: Cluster of orthologous groups
Ddx: Diadinoxanthin
DSP1: Death-specific protein
DTT: Dithiothreitol
Dtx: Diatoxanthin
ELIP: High light induced protein
ETR: Electron transport rate
FADS: Fatty acid desaturase
FAMEs: Fatty acid methyl esters
FCPs: Fucoxanthin Chl *a/c* proteins
FDR: False discovery rate
GO: Gene ontology
GOX: Glycolate oxidase
HL: High light
HPLC: High performance liquid chromatography
HSP: Heat shock proteins
IDA: Information-dependent acquisition
iTRAQ: Isobaric tags for relative and absolute quantification
LCFAL: Long-chain-fatty-acid-CoA ligase
LHC: Light-harvesting complex
LL: Low light
MsrA: Methionine sulfoxide reductase
NDSE: Nucleoside-diphosphate-sugar epimerase
NPQ: Non-photochemical quenching
PDI: Protein disulfide isomerase
PEPCase: Phosphoenolpyruvate carboxylases
PEPCK: Phosphoenolpyruvate carboxykinase
PGK: Phosphoglycerate kinase
PKK: Phosphoribulokinase
PPDK: Pyruvate phosphate dikinase
PPOM: Peptidoglycan-associated outer membrane protein
PSII: Photosystem II
RDS: Retinol dehydrogenases
ROS: Reactive oxygen species
Rubisco: Carboxylase/oxygenases
SRPS: Serine palmitoyltransferase
STI1: Stress-inducible protein
TAG: Triacylglycerol
TEAB: Tetraethylammonium bicarbonate
VDL: Violaxanthin de-epoxidase-like
XC: Xanthophyll cycle
