# Supplementary material for: High light stress triggers distinct proteomic responses in the marine diatom Thalassiosira pseudonana
Source: BMC Genomics. 2016 Dec 5;17:994. doi: 10.1186/s12864-016-3335-5 (PMC5139114; doi:10.1186/s12864-016-3335-5)
Supplement: Additional file 1: Table S1. — Primers used in this study for qRCR. (DOC 40 kb) [file 12864_2016_3335_MOESM1_ESM.doc]

| Gene | Accession number | Forward | Reverse |
| --- | --- | --- | --- |
| Glycolate oxidase | gi|220975250 | GATTGGGTTGAGATGGTATTGG | GGCGATTTCAGATTGGCAC |
| Fucoxanthin chlorophyll a/c protein, LI818 clade (Lhcx4) | gi|220974177 | TCGGATTCTCCAACGGTGA | TGGCAAGGAAGAATGGGAAG |
| Antibiotic biosynthesis monooxygenase | gi|220968047 | TTCAATGCTTGGCGACAGG | CATCCGTCAAAAGTTTCAGGG |
| Peroxiredoxin Q | gi|220975768 | GCATTCAAGGTTCCCAAGG | TTCTTAGCAGGCTTCATCGC |
| Fatty acid desaturase | gi|220973009 | GAAAAGAACCAAGGCGAAGC | AACCCCACCACAGGAATAATC |
| Fucoxanthin chlorophyll a/c protein, LI818 clade (Lhcx6) | gi|220969145 | GAGGCTCTTCCCAGTCCATT | TCCAGTTTCCAAAGTCAGGC |
| Chitinase | gi|220967856 | TTCGCCCCTGGAACTGATA | GGCAGAACGGCACCATAAA |
| Long-chain-fatty-acid-CoA ligase | gi|209585777 | ATCGCAGTATTGAACCCTCG | CGTTTGTTTCCTCGGATTTC |
| Actin | gi|220977426 | TGGATGTAGGCGAGGGTGTG | ATCAGCCTCAGCCGTAGTCG |

Table S1 Primers used in this study for qRCR.
