# Supplementary material for: High light stress triggers distinct proteomic responses in the marine diatom Thalassiosira pseudonana
Source: BMC Genomics. 2016 Dec 5;17:994. doi: 10.1186/s12864-016-3335-5 (PMC5139114; doi:10.1186/s12864-016-3335-5)
Supplement: Additional file 2: Figure S1. — Workflow for proteomic experiments using iTRAQ labeling in this study. T. pseudonana cells were acclimated in low light (LL) for 4 weeks and exposed to high light (HL) for 10 h. Three biological replicates were performed. The extracted proteins were reduced, alkylated, and digested with trypsin after cell pellets were lysed. The tryptic peptides were then labeled with iTRAQ reagents and put together as shown. The SCX chromatography was performed to fractionate peptides and fractions were collected. The eluted peptides were analyzed by LC-MS/MS. (PPT 61 kb) [file 12864_2016_3335_MOESM2_ESM.ppt]

## Slide 1
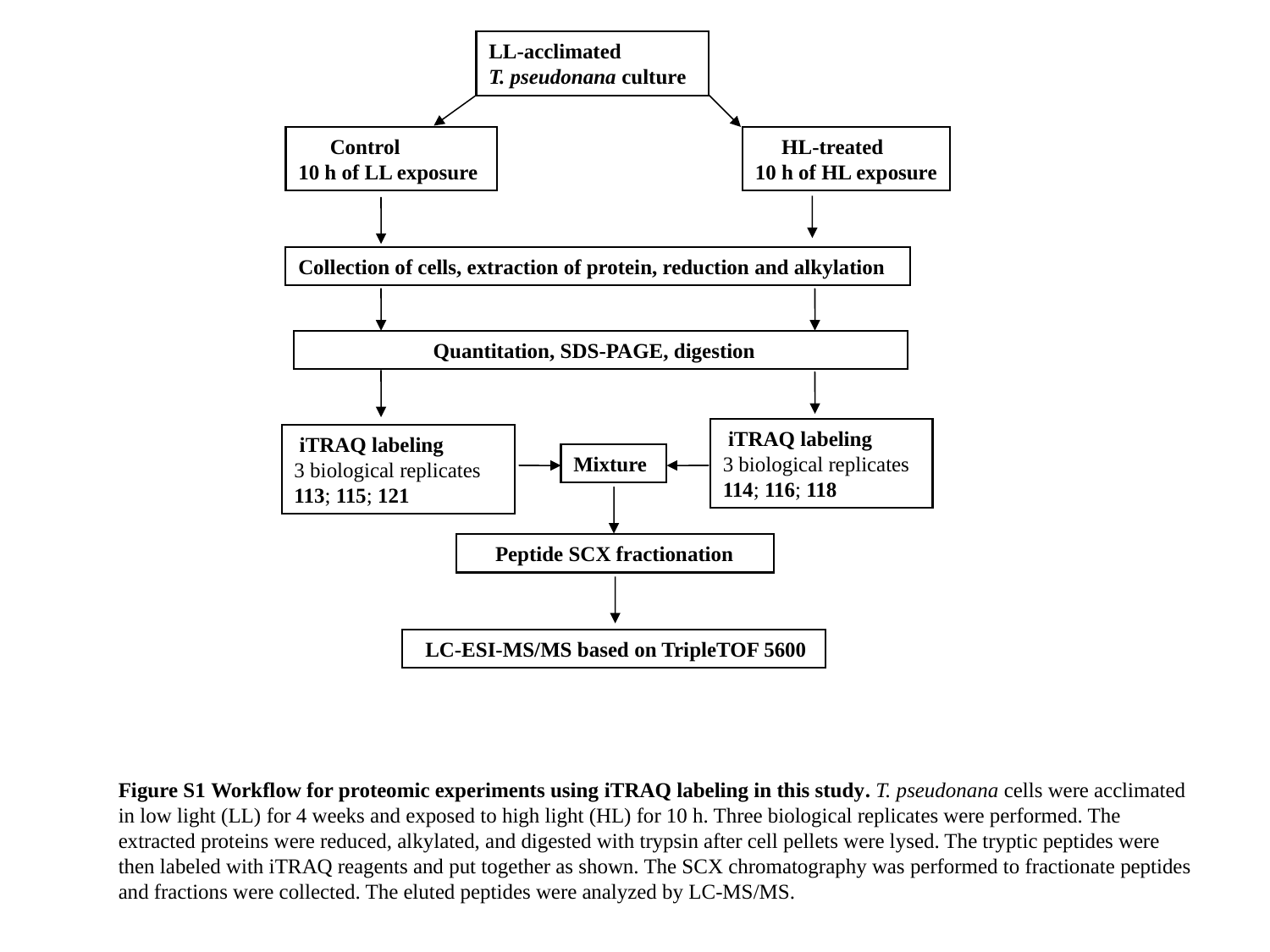

LL-acclimated
T. pseudonana culture
 Control
10 h of LL exposure
 HL-treated
10 h of HL exposure
Collection of cells, extraction of protein, reduction and alkylation
	Quantitation, SDS-PAGE, digestion
 iTRAQ labeling
3 biological replicates
114; 116; 118
 iTRAQ labeling
3 biological replicates
113; 115; 121
Mixture
 Peptide SCX fractionation
 LC-ESI-MS/MS based on TripleTOF 5600
Figure S1 Workflow for proteomic experiments using iTRAQ labeling in this study. T. pseudonana cells were acclimated
in low light (LL) for 4 weeks and exposed to high light (HL) for 10 h. Three biological replicates were performed. The extracted proteins were reduced, alkylated, and digested with trypsin after cell pellets were lysed. The tryptic peptides were then labeled with iTRAQ reagents and put together as shown. The SCX chromatography was performed to fractionate peptides and fractions were collected. The eluted peptides were analyzed by LC-MS/MS.
