# Supplementary material for: High light stress triggers distinct proteomic responses in the marine diatom Thalassiosira pseudonana
Source: BMC Genomics. 2016 Dec 5;17:994. doi: 10.1186/s12864-016-3335-5 (PMC5139114; doi:10.1186/s12864-016-3335-5)
Supplement: Additional file 4: Figure S2. — Error distribution (A, B, C, D, E, and F) among three biological replicates and CV distribution (G) between control and HL-treated samples. (DOC 250 kb) [file 12864_2016_3335_MOESM4_ESM.doc]

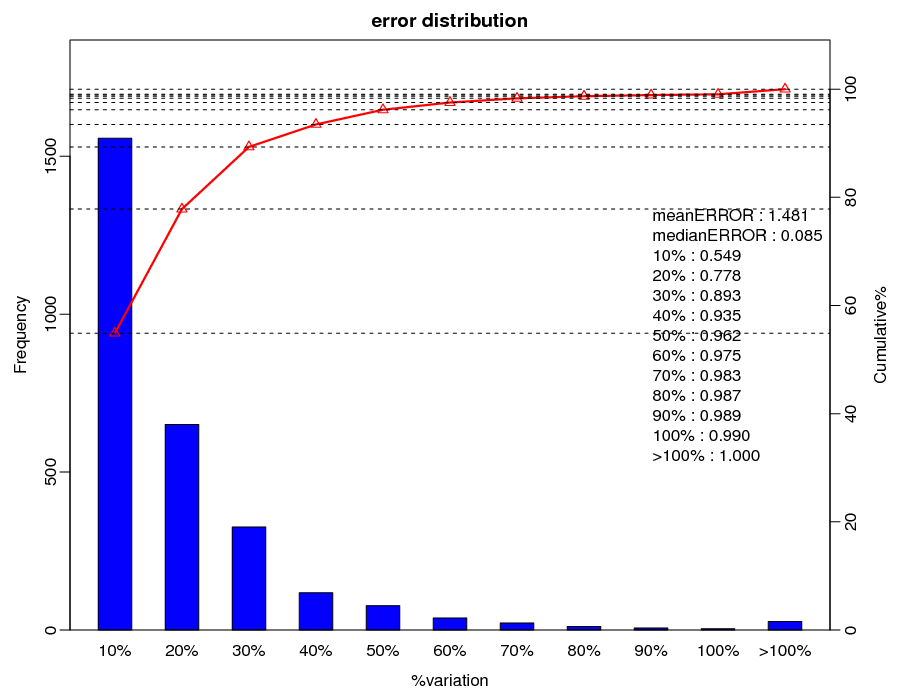

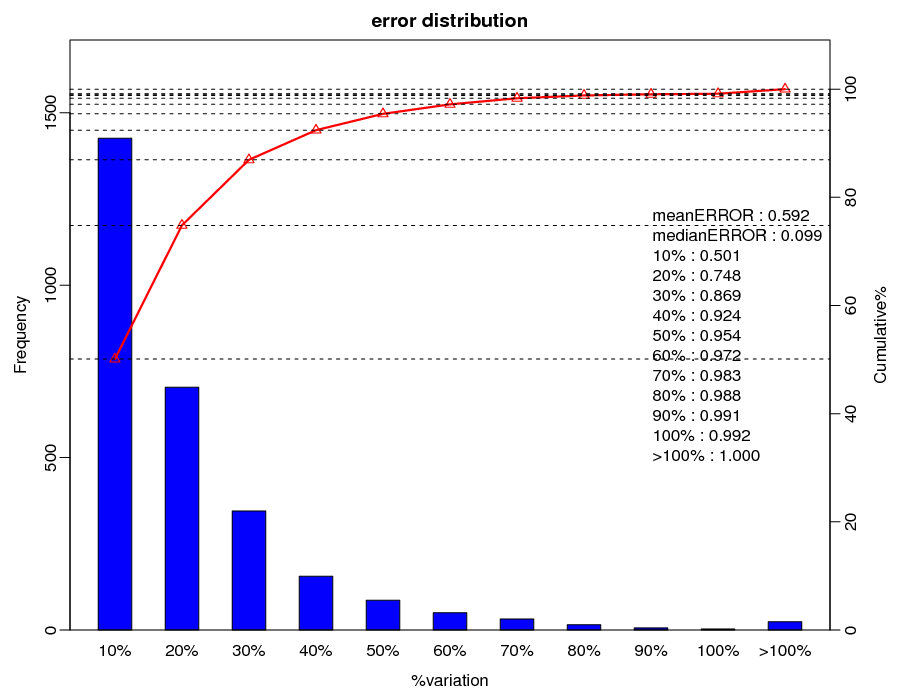


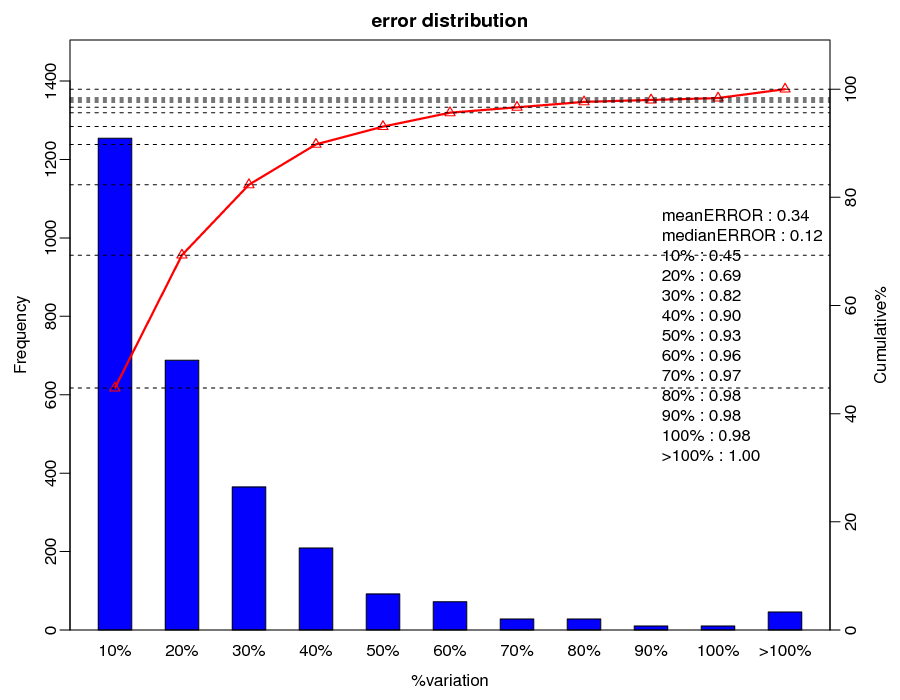
 A B


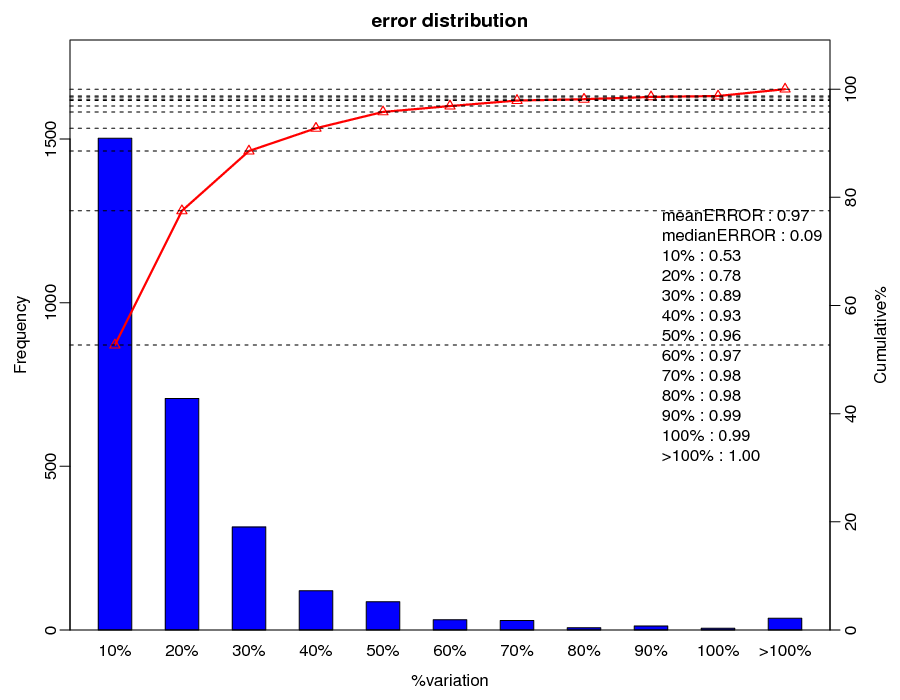


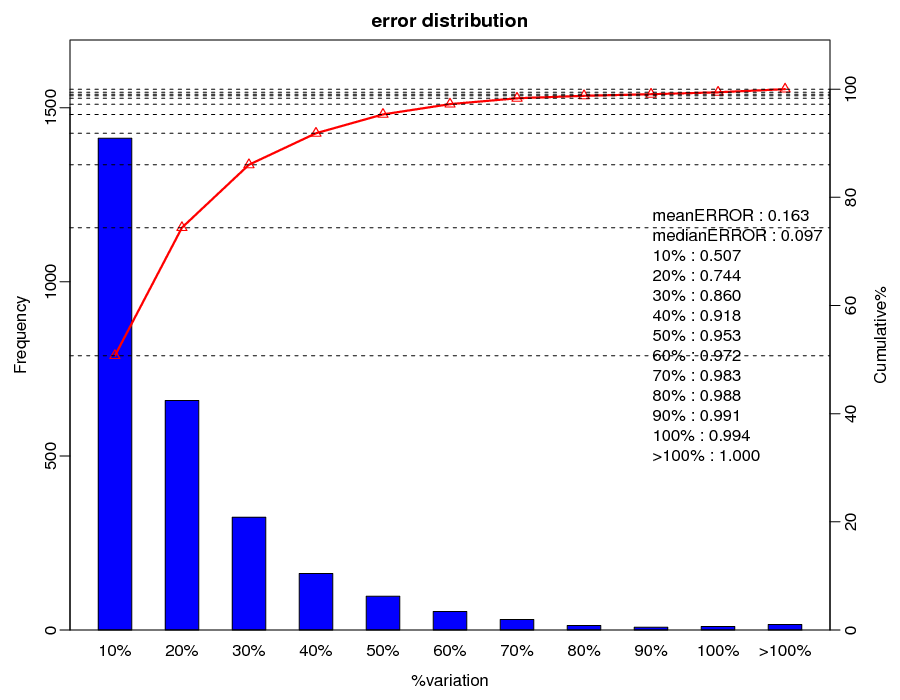
 C D


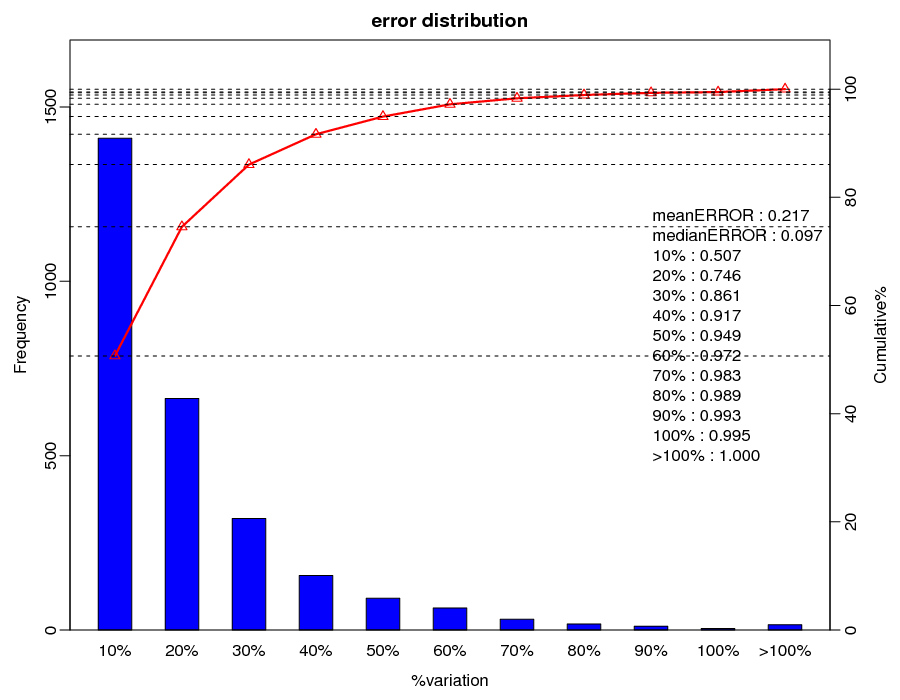


E F


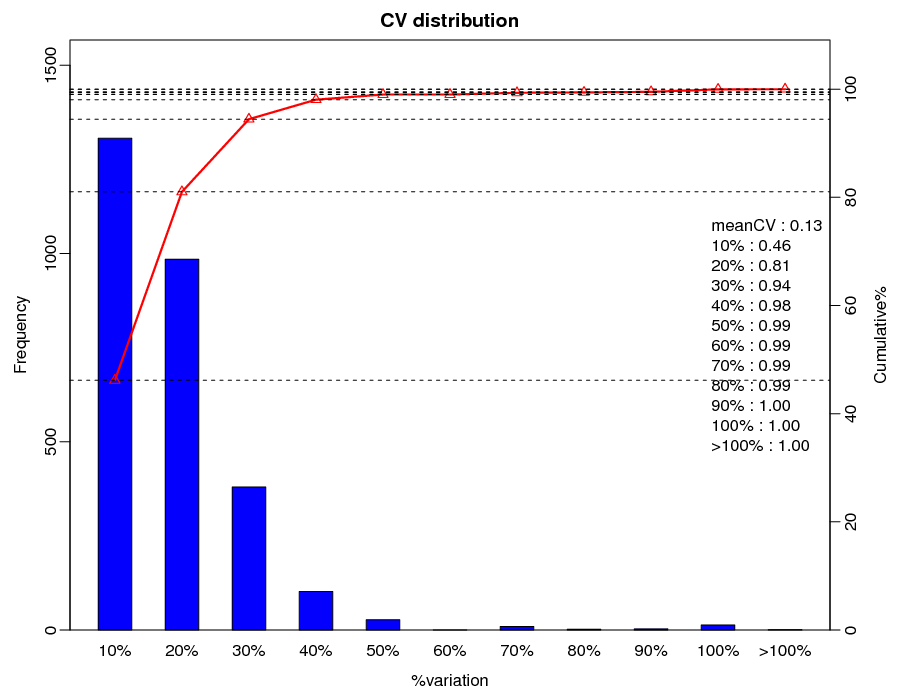


G

Figure S2 Error distribution (A, B, C, D, E, and F) among three biological replicates and CV distribution (G) between control and HL-treated samples.
